# Carbohydrate Mouth Rinse Fails to Improve Four-Kilometer Cycling Time Trial Performance

**DOI:** 10.3390/nu10030342

**Published:** 2018-03-12

**Authors:** Flávio O. Pires, Cayque Brietzke, Fabiano A. Pinheiro, Katherine Veras, Eugênia C. T. de Mattos, André L. F. Rodacki, Carlos Ugrinowitsch

**Affiliations:** 1 Exercise Psychophysiology Research Group, School of Arts, Science and Humanities, University of São Paulo, São Paulo 03828-000, Brazil; cayquebbarreto@usp.br (C.B.); fapcor@hotmail.com (F.A.P.); 2 Human Movement Science and Rehabilitation Program, Federal University of São Paulo, Santos 11015-020, Brazil; 3 School of Physical Education and Sport, University of São Paulo, São Paulo 05508-030, Brazil; ugrinowitsch@gmail.com; 4 Integrated Group of Biotechnology, Lab Adipose Tissue Biology, University of Mogi das Cruzes, São Paulo 05305-000, Brazil; katherine.veras@gmail.com; 5 Physical Education Department, Federal University of Paraná, Paraná 80060-000, Brazil; eugeniamattos@hotmail.com (E.C.T.d.M.); rodacki@ufpr.br (A.L.F.R.)

**Keywords:** central fatigue, peripheral fatigue, supplementation, twitch interpolation

## Abstract

We investigated if a carbohydrate (CHO) mouth rinse may attenuate global fatigue and improve 4-km cycling time trial (TT_4km_) performance. After a preliminary session, cyclists (*n* = 9) performed a TT_4km_ after a CHO or placebo (PLA) mouth rinse. Mean power output, time, and ratings of perceived exertion (RPE) were recorded throughout the TT_4km_. Twitch interpolation responses (%VA; voluntary activation and ∆Tw; delta peak twitch torque) were compared pre and post TT_4km_ with traditional statistics and effect size (ES) analysis. Time-to-complete the 4 km and mean power output were comparable between CHO (386.4 ± 28.0 s) and PLA (385.4 ± 22.4 s). A lower central (*p =* 0.054) and peripheral (*p =* 0.02) fatigue in CHO than in PLA were suggested by an extremely-large ES in %VA (manipulation main effect: *p* = 0.052, *d =* 1.18; manipulation-by-time interaction effect: *p* = 0.08, *d =* 1.00) and an extremely, very-large ES in ∆Tw (manipulation main effect: *p* = 0.07, *d =* 0.97; time-by-manipulation interaction effect: *p* = 0.09, *d =* 0.89). The RPE increased slower in CHO than in PLA (*p* = 0.051; *d =* 0.7). The apparent reduction in global fatigue (central and peripheral) and RPE_SLOPE_ with only one CHO mouth rinse were not translated into improved TT_4km_ performance. Further tests may be required to verify if these likely differences in global fatigue might represent an edge in the short-lasting cycling time trial performance.

## 1. Introduction

Carbohydrate (CHO) mouth rinse has been used to improve performance in long- (i.e., >45 min) [1,2] and short-lasting (i.e., <45 min) [3] cycling time trials. The most probable mechanism involves oral cavity CHO sensors-activated brain areas, as a functional magnetic resonance imaging (*f*MRI) study verified greater activation in areas such as orbitofrontal cortex, insula and operculum frontal when individuals rinsed their mouths with a CHO solution [4]. This same study suggested that the greater activation in these cerebral areas was associated with the improved 1 h cycling trial performance observed when those same participants were evaluated in a different testing session. Accordingly, an increased motor output as a result of an improved cerebral activation has been suggested, as the CHO mouth rinse increased corticomotor pathway excitability as indicated by a higher motor evoked potential [5]. Therefore, overall suggestion assumes that improvements in cycling time trial performance after CHO mouth rinse may be related to ameliorated neural drive.

Evidence has suggested the use of CHO mouth rinse as a strategy to improve cycling time trial performance. Ataide-silva et al. [3] observed increases in central drive (as measured by electromyography (EMG)) and 20-km cycling time trial performance when participants rinsed their mouth with a CHO solution, after starting the trial in a fasting state and with low muscle glycogen stores. Interestingly, such a result was not confirmed when they started the trial either in a fed or in a fasted state with normal muscle glycogen content, thereby indicating that potential CHO mouth rinse effects may depend on these features. In fact, this topic has become controversial as some have failed to show increases in performance even under a muscle glycogen-reduced condition [6], while others have found that CHO mouth rinse may potentiate cycling performance in both fasting and fed states [2]. Furthermore, the fact that some have found positive effects in cycling performance after CHO mouth rinse, while others have shown that attenuated central fatigue after CHO mouth rinse did not translate into improved performance [7], has challenged the use of CHO mouth rinse to potentiate cycling performance.

Despite equivocal results, practitioners, athletes, trainers and coaches may be interested in potential benefits of CHO mouth rinse, since a qualitative cycling study reported that the more committed cyclists to the sport the more the pressure to use supplements to improve performance [8]. In this perspective, cyclists of different competitive levels make use of nutritional supplements to improve performance, and anecdotal data suggest that recreational cyclists are among the group of individuals that have frequently used this strategy to improve performance on both training and competition settings. To the best of our knowledge, however, only a few studies have focused on the potential benefits of CHO mouth rinse during typical cycling time trials [3,9]. Accordingly, most of the studies have focused on 1-h time trials [2,6,10] instead of distance-based ones such as 4 km, 20 km and 40 km, which would properly resemble competition scenarios.

In this regard, a recent study verified that CHO mouth rinse did not improve 40-km cycling time trial performance under hyperthermia [11], while others reported otherwise in 20-km cycling time trials performed in normothermia [3,9]. However, there is paucity of data on the effects of CHO mouth rinse on shorter-lasting trials such as 4 km (TT_4km_). Although most cycling literature has focused on long- (i.e., 40 km and 20 km) [3,9,11,12] as well as short-lasting time trials (TT_4km_) [13,14] when investigating manipulations to potentiate cycling performance, evidence for the potential benefit of the CHO mouth rinse in short-lasting cycling time trial performance has yet to be provided. For example, a previous study reported that CHO mouth rinse attenuated global fatigue in an endurance cycling trial, despite the unchanged performance. Most importantly, central as well as peripheral fatigue were attenuated when cyclists and triathletes rinsed their mouth with CHO solution [7]. However, whether CHO mouth rinse may attenuate both peripheral and central fatigue (i.e., global fatigue), thus further potentiating TT_4km_ performance, is currently unknown. 

Therefore, this study was designed to determine if CHO mouth rinsing could potentiate performance in a short-lasting cycling time trial (i.e., TT_4km_). We hypothesized that CHO mouth rinse will attenuate both peripheral and central fatigue, thus potentiating TT_4km_ performance. It is important to mention that all assessments were performed under a fed state, as it is a regular condition met by most athletes during training sessions and competitions.

## 2. Materials and Methods 

### 2.1. Participants

Nine male recreational cyclists (36.9 ± 6.4 years; 73.1 ± 13.0 kg; 180.0 ± 5.0 cm; maximal oxygen uptake (VO_2MAX_) of 54.3 ± 9.3 mL·kg^−1^·min^−1^; peak power output (W_PEAK_) of 333.4 ± 28.6 W) with some experience in cycling time trials (~2 years taking part in regional competitions), participated in this study. This study was carried out in accordance with the Declaration of Helsinki, being previously approved by the University of São Paulo’s Ethics Committee (#0023.0.342.000-10). All participants were informed about the experimental procedures, before giving the signature on a written consent form. They were free from cardiovascular and musculoskeletal disorders or other comorbidities that could affect dependent variables. 

### 2.2. Study Design

This study consisted of four visits in order to: (1) familiarize participants with the Borg’s scale (1982) [15], TT_4km_ and twitch interpolation technique; (2) perform a maximal incremental test (MIT) to assess VO_2MAX_ and W_PEAK_, familiarize them with the twitch interpolation technique and to collect an alimentary record; (3) perform a TT_4km_ after rinsing the mouth with a CHO or placebo (PLA) solution (sessions 3 and 4). Visits 1 and 2 followed a sequential order, while 3 and 4 were performed in a double-blind, counterbalanced fashion, after random designation. The twitch interpolation technique was applied before and immediately after the TT_4km_. Participants were instructed to refrain from intense physical exercises for 72 h and from consumption of coffee, alcoholic and energy beverages for 24 h before the sessions.

### 2.3. Dietary Control

In practical situations, athletes usually ingest a balanced diet approximately 2 h before the training sessions or competitions. Therefore, we evaluated cyclists in a fed state, without promoting a glycogen-reduced content, as we were interested to verify if the CHO mouth rinse may work as an ergogenic aid in practical situations. Then, from the data provided by their dietary habits, a qualified nutritionist prepared individualized diets (~55% CHO, ~25% protein and ~20% fat), which was replicated during the 24 h preceding the sessions 3 and 4. Participants were strongly oriented to follow the individualized food diary in the day before the test sessions, and to consume a standard breakfast 2 h before the onset of the procedures, after overnight fasting. Participants confirmed they followed these procedures accordingly (i.e., fed state) upon arrival at the laboratory.

### 2.4. CHO Mouth Rinse Manipulation

Participants were given a 25 mL solution of either CHO or PLA before the trial. They rinsed the solution around their mouth for 10 s during the pace-controlled warm up routine, then they spat it into a bowl. Importantly, we used a 10 s CHO mouth rinse because a previous study had shown that a 10 s mouth rinse significantly improved cycling performance when compared to 5 s [16]. The volume spat into the bowl was checked in order to confirm no ingestion of CHO solution. The CHO solution was made by diluting 64 g of glucose per 1000 mL of water, whilst PLA solution was made by diluting 36.2 g of a non-caloric sweetener containing saccharin in 1000 mL of water, in accordance with previous study [10]. Cyclists rinsed their mouth only once, during the controlled-pace warm up, as the TT_4km_ is a short trial completed under maximal effort, comparable to a MIT [17]. Consequently, the nature of the TT_4km_ (i.e., vigorous body movement and hyperventilation) prevented us to include mouth rinses during exercise without compromising the experimental control, thereby ensuring that participants would not ingest the solutions during the trial.

### 2.5. Four Kilometers Cycling Time Trial

Cyclists complete the TT_4km_ with their own bike attached to a cycle-simulator (Computrainer™ Lab 3D, RacerMate, Seattle, WA, USA). After a 5 min self-paced warm up, cyclists warmed up 2 min at a controlled-pace warm up (80 rpm; 100 W); then they immediately started the TT_4km_ from a starting power output corresponding to 100 W. Cyclists were asked to complete the TT_4km_ as fast as possible, having available feedback of power output, pedal cadence, distance and time throughout the trials.

### 2.6. Procedures, Measures and Data Analysis

#### 2.6.1. Voluntary Activation and Contractile Properties

Pre- and post-TT_4km_ knee extension torque assessments (interpolated torque and twitch torque) were performed on an isokinetic dynamometer (Biodex System 3, Biomedical Systems, Newark, CA, USA) on the right leg (i.e., dominant limb). Participants were seated on the device’s chair with their hips at 90° and knees at 60° from the horizontal. Their chest and hips were fixed with Velcro straps to avoid accessory movements. The estimated center of rotation of the knee joint was aligned with the dynamometer center of rotation and knee extension torque was assumed to be equal to the torque produced by the Biodex motor, corrected by gravity. 

Supramaximal electrical stimulus was applied through two 7.5 × 13 cm self-adhesive electrodes (Mundinter Dantec Clavis^®^, Lisbon, Portugal) positioned on the first (cathode) and second thirds (anode) of the distance from the anterior superior iliac spine (ASIS) to the proximal border of the patella of the right thigh, respectively. The thigh was shaved, abraded, and cleaned with an isopropyl alcohol pad inside gauze to reduce skin impedance before electrode placement. Two rectangular pulses of 1 ms were delivered at 100 Hz over the muscle belly (Nicolet Viking Quest portable EMG apparatus; CareFusion^®^, San Diego, CA, USA) with the intensity of stimulation individually determined during the maximum torque obtained at rest [18]. The maximum torque test was performed having the participants tested according to the manufacturer guidelines. They were instructed to perform a maximum isometric knee extension for 5 s. The electrical stimuli were applied visually at a point of a plateau of maximal torque production [19]. A second stimulus was delivered 1.5 s after the end of contraction (control twitch). The torque values were controlled by visual feedback, and saved for posterior analysis (LabVIEW 2010, National Instruments, São Paulo, Brazil). VA was calculated through the maximum torque obtained with interpolated stimuli (interpolated twitch) during maximal voluntary contraction (MVC) with the control twitch (contraction at rest), according to the Equation (1) [20,21]. The difference in peak twitch torque (∆Tw) obtained through potentiated 100 Hz doublet at rest (after the MVC), from pre- to post-cycling trial, indicated the change in peripheral fatigue [7,22].

(1) $$VA\;\left(\%\right)=\left[1-\left(\frac{interpolated\;twitch}{control\;twitch}\right)\right]\times 100$$

#### 2.6.2. Performance and Pacing Strategy

The TT_4km_ performance was determined as the time to complete the trial. Power output data was averaged over each 0.4 km and plotted as a function of the distance, thus allowing pacing strategy assessments. Furthermore, the mean power output recorded throughout the trial was calculated (W_MEAN_).

#### 2.6.3. Ratings of Perceived Exertion

The ratings of perceived exertion (RPE) was measured every 1 km, thereafter these data were plotted as a function of the distance to obtain the slope of the RPE-distance relationship (RPE_SLOPE_).

### 2.7. Statistical Analysis

The data was initially checked for Gaussian distribution through the Shapiro-Wilk test. Results of the twitch interpolation technique (i.e., VA and ∆Tw) were compared between pre and post TT_4km_ by using a 2 × 2 mixed models, having time (pre vs. post) and mouth rinse (CHO vs. PLA) as fixed factors and subjects, as the random one. Pacing strategy was further compared through mixed models, having distance (i.e., 0.4 km, 0.8 km and 4 km) and mouth rinse as fixed factors, subjects were the random factor. Bonferroni correction was used for multiple comparisons, when F values were significant. Time to complete the trial, W_MEAN_ and RPE_SLOPE_ during TT_4km_ between CHO and PLA mouth rinse were compared through a paired Student-t test. Statistical significance was set at *p* < 0.05 (v. 22 SPSS software, IBM, New York, NY, USA), being considered as borderline *p*-values when 0.05 < *p* < 0.1. Importantly, we did not perform a priori power calculation, as this would require a known coefficient of variation, magnitude of the treatment effect and pre-post correlations for CHO mouth rinse interventions in short time trials such as 4 km. This information is not available, as is the first study investigating the effects of CHO mouth rinse in TT_4km_ performance. Thus, we chose to have a sample size similar to most of the studies on CHO mouth rinse; therefore, we initially included 12 cyclists. However, three dropped out due to personal reasons so that 9 cyclists concluded the study. We performed a post-hoc power analysis, which indicated the actual power to obtain a type II error rate. In addition, we calculated the effect size (ES) as an alternative, more intuitive approach than inferential statistics [23]. ES was initially calculated according to the family’s test (i.e., Cohen’s d for t-tests and ƞ^2^ for *F*-tests) and subsequently converted to Cohen’s d (to make comparisons with previous studies possible), being interpreted as proposed by Hopkins et al. [23], as small (<0.1), moderate (0.1> and <0.3), large (0.3> and <0.5), very large (0.5> and <0.9) and extremely large (>0.9). 

## 3. Results

### 3.1. Effects of CHO Mouth Rinse on TT_4km_ Performance

**Figure 1:**
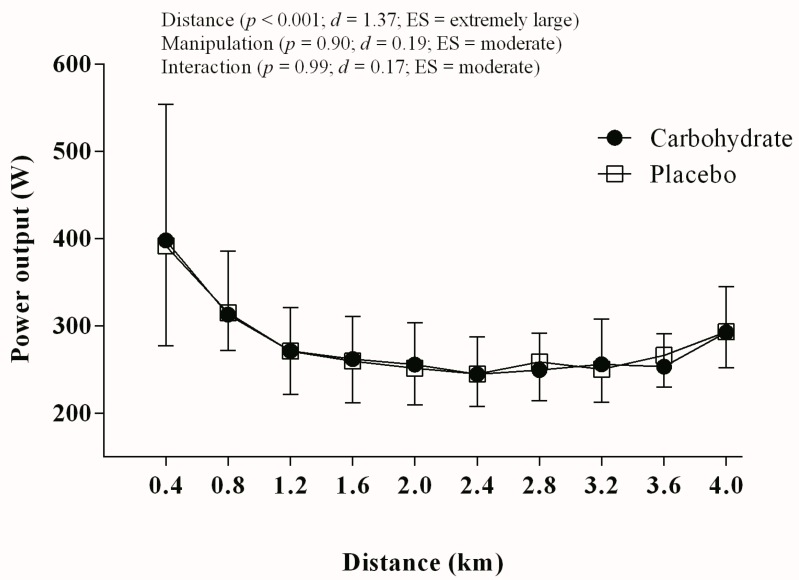
Power output throughout the TT_4km_ in carbohydrate (filled circles) and placebo mouth rinse (open squares).

Performance in TT_4km_ did not improve when cyclists rinsed the mouth with CHO as the time to complete the trial remained unchanged relative to PLA (386.4 ± 28.0 s vs. 385.4 ± 22.4 s, respectively; *p* = 0.85; *d* = 0.04, ES small; power = 0.85). Accordingly, W_MEAN_ remained comparable between CHO and PLA conditions (275.4 ± 43.3 W vs. 277.0 ± 35.0 W, respectively; *p* = 0.83; *d* = 0.04, ES small; power = 0.80). In mixed models, a distance main effect was detected so that the power output changed along the trial (*p* < 0.001; *d* = 1.37, ES extremely large, power = 0.84). However, neither manipulation main effect (*p* = 0.90; *d* = 0.19, ES moderate; power = 0.90) nor manipulation by distance interaction effect (*p* = 0.99; *d* = 0.17, ES moderate; power = 0.90) was found (Figure 1).

### 3.2. Effects of CHO Mouth Rinse on MVC, Central and Peripheral Fatigue, and RPE Responses

**Figure 2:**
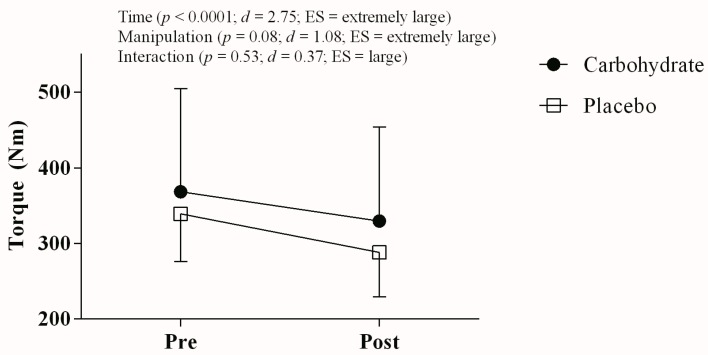
Torque responses measured before and after TT_4km_ in carbohydrate (filled circles) and placebo mouth rinse (open squares).

There was no significant interaction effect for MVC (*p* = 0.53; *d* = 0.37; ES large; power = 0.63), but there was a significant main time effect (*p* < 0.0001; *d* = 2.75; ES very large; power = 0.57). Additionally, borderline *p*-values together with extremely large ES (*p* = 0.08; *d* = 1.08; ES extremely large; power = 0.77) were found for manipulation main effect (Figure 2). There was no time main effect for VA (*p* = 0.28; *d* = 0.59; ES very large; power = 0.57). However, borderline *p*-values were observed together with extremely large ES for manipulation main effect (*p* = 0.052; *d* = 1.18, ES extremely large; power = 0.88) and time by manipulation interaction effect (*p* = 0.08; *d* = 1.00, ES extremely large; power = 0.76), thus suggesting that VA (i.e., central activation) after TT_4km_ was greater in CHO than in PLA mouth rinse (*p* = 0.054). Changes in VA were similar between CHO mouth rinse (91.56 ± 3.42 vs. 90.42 ± 7.47; Δ −1.25%) and PLA mouth rinse (83.97 ± 13.57 vs. 82.96 ± 13.24; Δ −1.20%).

**Figure 3:**
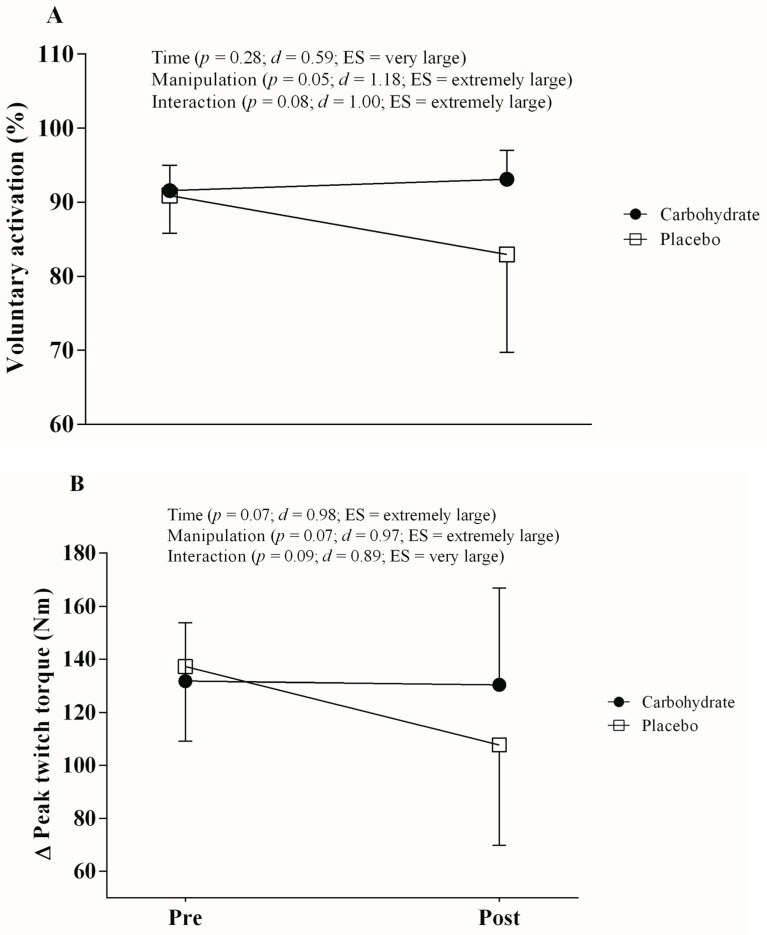
Twitch interpolation responses such as voluntary activation (panel **A**) and delta peak twitch torque (panel **B**) measured before and after TT_4km_ in carbohydrate (filled circles) and placebo mouth rinse (open squares).

Regarding ∆Tw responses, there were also borderline *p*-values together with extremely large ES for time main effect (*p* = 0.07; *d* = 0.98; ES extremely large; power = 0.68) and manipulation main effect (*p* = 0.07; *d* = 0.97; ES extremely large; power = 0.67). Accordingly, we observed a borderline *p*-value together with a very large ES for time by manipulation interaction effect (*p* = 0.09; *d* = 0.89; ES very large, power = 0.60), thereby suggesting a lower peripheral fatigue level after TT_4km_ in CHO than in PLA mouth rinse manipulation (*p* = 0.02). Reductions in the ∆Tw occurred at a lower magnitude in CHO mouth rinse (131.73 ± 21.98 vs. 130.35 ± 36.48 N; Δ −1.05%) than in PLA (137.17 ± 28.05 vs. 107.71 ± 37.89 N; Δ −21.48%) pre to post, respectively. Responses of twitch interpolation are displayed in Figure 3 (panels A and B).

**Figure 4:**
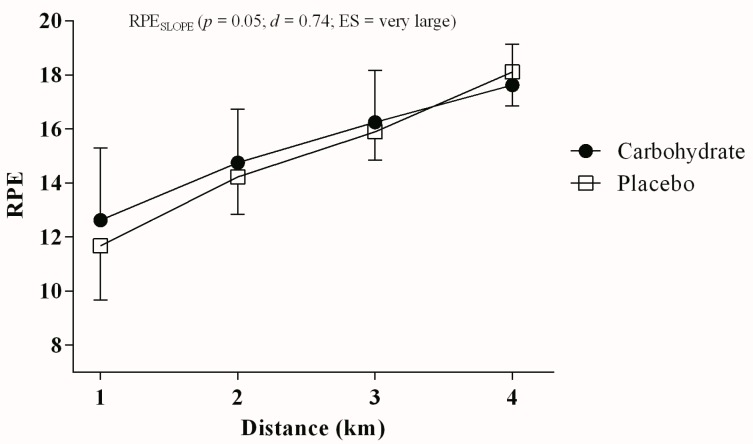
RPE responses measured each 1 km during the TT_4km_ in carbohydrate (filled circles) and placebo mouth rinse (open squares).

Cyclists perceived RPE rising steeper (higher RPE_SLOPE_) in PLA (2.04 ± 0.53) than in CHO mouth rinse (1.65 ± 0.53) (*p* = 0.051; *d* = 0.7; ES very large, power = 0.80, Figure 4).

## 4. Discussion

This is the first study investigating the effects of CHO mouth rinse on TT_4km_ performance. Although the CHO mouth rinse seemed to mitigate central and peripheral fatigue, it was not effective in improving TT_4km_ performance in the laboratory. These results challenge the suggestion to use CHO mouth rinse as a strategy to improve performance in short-lasting cycling time trials.

The CHO mouth rinse was ineffective to improve TT_4km_ performance, as PLA and CHO conditions showed similar W_MEAN_ and time elapsed to complete the trial. Similarly, pacing strategy did not differ between conditions (i.e., CHO mouth rinse and PLA) throughout the trial. Despite several studies [1,3,24] showing that CHO mouth rinse is effective to improve cycling performance, our results corroborate those of others showing no benefits in using this strategy [7,9,25,26]. Some aspects could be highlighted to discuss this controversy. It is likely that the effects of CHO mouth rinse on performance are regulated by central factors, as an earlier *f*MRI study reported that the enhanced CHO mouth rinse-triggered reward circuit activation was followed by a greater activation in frontal, motor planning areas [4,27]. An additional study also found through transcranial magnetic stimulation that maximal voluntary force and motor evocated potential were improved with CHO mouth rinse, indicating improved sensorimotor integration [5]. Thus, it has been suggested that CHO mouth rinse improves central motor drive and reduces central fatigue [3,28]. However, Thomas et al. [22] observed that the magnitude of central fatigue was lower, and peripheral fatigue was greater in TT_4km_ (~6 min) than in longer-lasting trials such as 20 km (~31.8 min) and 40 km (~65.8 min), which may have precluded the improvement in performance observed herein. Therefore, long (i.e., TT_20km_ and TT_40km_) rather than short cycling trials (i.e., TT_4km_) should benefit from this ergogenic strategy [1,2,4].

Despite the lack of statistical significance in our twitch interpolation data, qualitative ES analyses suggest that CHO mouth rinse may hamper central fatigue development when compared to PLA (extremely large ES, *p* = 0.08). Nevertheless, the attenuated decrease in central drive with CHO mouth rinse did not seem to change performance indices (i.e., time to completion and average power). Interestingly, qualitative ES analysis also suggested that CHO mouth rinse further attenuated the occurrence of peripheral fatigue, as the very large ES indicated that pre-to-post reductions in ∆Tw occurred at a lower magnitude in CHO than in PLA (*p* = 0.09). Surprisingly, as reported elsewhere, the very large ES for a positive influence of CHO mouth rinse on peripheral fatigue was not translated into improved cycling performance [7,26,29]. The attenuation in peripheral fatigue due to CHO mouth rinse is hard to reconcile, however, one may suggest that an ameliorated neuromuscular recruitment due to the CHO mouth rinse [3] may have led to less excitatory inputs required from motor cortices at the same motor output [30]. Taken together, results of the present study suggest that CHO mouth rinse was ineffective to improve TT_4km_ performance in the laboratory. However, one may suggest that the likely reduced central and peripheral fatigue observed herein may represent an edge in training sessions combining multiple exercise bouts, as the attenuated global fatigue with CHO mouth rinse may play a role to improve recovery between exercise bouts. Future studies are required to confirm this hypothesis.

In line with a lower global fatigue in CHO mouth rinse during TT_4km_, cyclists presented a smaller RPE_SLOPE_ in CHO mouth rinse than in PLA. Similarly, Jeffers et al. [7] assessed fatigue mechanisms on a cycling time trial (15 min) preceded by a preload, CHO or PLA mouth rinse. They found that CHO mouth rinse did not improve performance in cycling time trials, although attenuating global fatigue [7]. It is possible that manipulations able to increase excitability between frontal and motor cortices could reduce the neural drive to skeletal muscles when generating the same motor output, thereby also reducing corollary discharges to the RPE [30]. Thus, the lower RPE_SLOPE_ observed in the CHO mouth rinse condition may also represent an edge in time trial competitions, as the slower increase in RPE throughout the trial may allow cyclists to improve the spurt at the end of a race. However, future studies investigating real competitions are required to confirm this, as we found no improvements in a TT_4km_ performed in the laboratory.

### 4.1. Methodological Aspects

Most studies investigating CHO mouth rinse used rinses at every 12.5% of total test duration. In contrast, we attempted to resemble practical situations, rinsing the mouth only at the beginning of the trial, as rinsing the mouth during a short time trial (i.e., TT_4km_) may impair performance, as cyclists are very likely to disengage from maximal effort while rinsing their mouth and spitting out. Additionally, there would be difficulties to ensure that they would not swallow the solution when completing the TT_4km_, since this trial is performed at effort levels compatible to maximal effort levels [17]. Hence, considering that most studies showing positive CHO mouth rinse effects on performance have used mouth rinses at regular intervals, it is possible that the single 10 s CHO mouth rinse at the beginning of the trial has been insufficient to potentiate performance, as effects of CHO mouth rinse seems to be immediate and timely [5]. In fact, it has been documented that cerebral activation is immediate at the time the subject starts the rinse [5], so that one may argue that the CHO mouth rinse would have likely required to be repeated throughout the trial to induce positive effects on TT_4km_ performance. However, we must point out that most studies reporting positive CHO mouth rinse effects on cycling performance repeated the mouth rinse every 12.5% of a 1-h cycling trial [1,4], thereby representing one CHO mouth rinse every ~450 s. In contrast, we used a single CHO mouth rinse prior to a TT_4km_ that lasted ~380 s, thus challenging the applicability of this strategy in short, high intensity cycling trials.

Although some studies have shown benefits of CHO mouth rinse on performance in both fed and fasted states [2,31], some showed improvements only if participants commenced the exercise in a fasted state (>4 h fasting) [3,24] and others failed to show improvements with CHO mouth rinse in a fed state [25,26], thereby leading to inconclusive interpretations about the influence of the pre-exercise fasting period. There is a questionable applicability of this intervention in real-world situations, as athletes (recreational or professional) usually avoid to train and compete in a fasting state.

According to Jeukendrup [32], the lack of statistical power can also be a limiting factor to find significant effects on performance data. Although we acknowledge that our sample size may have limited inferential power (mainly those related to the twitch interpolation technique) the sample size investigated in the present study (i.e., nine participants) was similar to that used by others, who observed similar results [29]. In fact, we are unware of exercise performance studies using the twitch interpolation technique with a number of participants that is much higher than nine [7,33]. Importantly, we emphasize that we did not perform a prior power calculation, as this would require a known coefficient of variation, magnitude of intervention effect, and pre-post correlations. Such information is usually not available, mainly for new research topics, as this may take long until the scientific literature consistently provide these data. This may be the case when considering CHO mouth rinse in short time trials such as 4 km. Thus, we decided not to perform a priori power calculation, as we would have not known how estimating the coefficient of variation and magnitude from different CHO mouth rinse studies (i.e., studies using longer time trials or different exercise modes) match those in TT_4km_. Furthermore, in the present study, we also used qualitative ES analysis as an alternative, more practical approach to determine the magnitude of the observed effects [23]. In this perspective, this analysis was important to show that borderline, non-significant *p*-values found in the present study (i.e., 0.05 < *p* < 0.1) would have been “statistically significant” with addition of a few participants, as suggested by very large and extremely large ES results. 

### 4.2. Practical Applications

The present study has a practical applicability and provides some insights. Regardless of training status, cyclists regularly use TT_4km_ as a mean to train for short distance cycling. Moreover, anecdotal data have suggested the use of CHO mouth rinse as a strategy to improve exercise performance in real training sessions and competitions, even in short, high intensity cycling. However, the CHO mouth rinse strategy appeared to have no effect on TT_4km_ performance, although global fatigue had been attenuated. Therefore, the present results challenge the use of CHO mouth rinse as a potential ergogenic aid for short, high intensity exercises. However, further tests should be performed to verify if these likely differences in global fatigue might represent an edge in training sessions combining multiple exercise bouts, or in the endspurt of a short-lasting cycling race.

## 5. Conclusions

The present study provided evidences that CHO mouth rinse is not an effective ergogenic strategy to improve performance in TT_4km_, thus challenging the use of CHO mouth rinse to potentiate performance in short time trials.
